# Ocean Acidification Affects Hemocyte Physiology in the Tanner Crab (*Chionoecetes bairdi*)

**DOI:** 10.1371/journal.pone.0148477

**Published:** 2016-02-09

**Authors:** Shannon L. Meseck, Jennifer H. Alix, Katherine M. Swiney, W. Christopher Long, Gary H. Wikfors, Robert J. Foy

**Affiliations:** 1 National Ocean and Atmospheric Administration, National Marine Fisheries Services, Northeaster Fisheries Science Center, Milford, Connecticut, United States of America; 2 Kodiak Laboratory, Resource Assessment and Conservation Engineering Division, Alaska Fisheries Science Center, National Marine Fisheries Service, NOAA, Kodiak, Alaska, United States of America; GEOMAR Helmholtz Centre for Ocean Research Kiel, GERMANY

## Abstract

We used flow cytometry to determine if there would be a difference in hematology, selected immune functions, and hemocyte pH (pH_i_), under two different, future ocean acidification scenarios (pH = 7.50, 7.80) compared to current conditions (pH = 8.09) for *Chionoecetes bairdi*, Tanner crab. Hemocytes were analyzed after adult Tanner crabs were held for two years under continuous exposure to acidified ocean water. Total counts of hemocytes did not vary among control and experimental treatments; however, there were significantly greater number of dead, circulating hemocytes in crabs held at the lowest pH treatment. Phagocytosis of fluorescent microbeads by hemocytes was greatest at the lowest pH treatment. These results suggest that hemocytes were dying, likely by apoptosis, at a rate faster than upregulated phagocytosis was able to remove moribund cells from circulation at the lowest pH. Crab hemolymph pH (pH_e_) averaged 8.09 and did not vary among pH treatments. There was no significant difference in internal pH (pH_i_) within hyalinocytes among pH treatments and the mean pH_*i*_ (7.26) was lower than the mean pH_e_. In contrast, there were significant differences among treatments in pH_i_ of the semi-granular+granular cells. Control crabs had the highest mean semi-granular+granular pH_i_ compared to the lowest pH treatment. As physiological hemocyte functions changed from ambient conditions, interactions with the number of eggs in the second clutch, percentage of viable eggs, and calcium concentration in the adult crab shell was observed. This suggested that the energetic costs of responding to ocean acidification and maintaining defense mechanisms in Tanner crab may divert energy from other physiological processes, such as reproduction.

## Introduction

Marine crustaceans inhabit environments with numerous temporal environmental variations (e.g., tidal, daily, and seasonal changes in temperature, pH, and salinity) that can influence metabolism, growth, molting, and survival [1,2]. Johnson [3] suggested that environmental variations may cause physiological stress that lead to changes in the mitotic activity of haematopoietic tissues resulting in changes in the turnover rate of hemocytes. In crustaceans, as in other invertebrates, hemocytes are circulating cells within the hemolymph that mediate immune defense functions [4,5]. Hemocytes can respond to foreign cells such as pathogens and parasites by phagocytosis or encapsulation and lysis [4,6,7]. In crustaceans, total hemocyte numbers can vary greatly when an individual is exposed to different environmental conditions (e.g., high temperature, low oxygen) [3,8,9] and invasion of bacteria, fungi, and viruses [10–12]. Within crustaceans there are three, morphologically-different classes of hemocytes: hyalinocyte cells (HC), semi-granular cells (SGC), and granular cells (GC) [4,13]. Each of the three cell types has different morphological features: hyalinocytes are the smallest cells and lack granules; semi-granular cells have small, intracellular granules; and granular cells have abundant cytoplasmic granules [14,15]. Each hemocyte cell type is believed to be responsible for different functions in immunity. For example, hyalinocytes are thought to be responsible for producing lectins to distinguish between self and non-self particles [12], semi-granular cells appear to have a role in encapsulation [16], and granular cells participate in the prophenoloxidase (proPO) system [17], which regulates melanization of pathogens and damaged tissues. Changes in the characteristics of hemocytes (i.e., viability, phagocytosis) can indicate physiological stress in crustaceans [18–20]. For example, exposing crustaceans to trace metals can decrease the number of circulating hemocytes [21,22]. Le Moullac and Haffner [18] found that ammonia in the environment resulted in a decrease in the number of hemocytes, with phagocytosis diminished, thus affecting the immune status of the shrimp *Penaeus stylirostris*. Even small chemical changes, such as salinity, in an estuary can have effects on total hemocyte numbers and phagocytosis [23,24].

Ocean acidification is an environmental change that may have substantial effects on marine animals. The global average surface pH has dropped 0.1 units since the industrial revolution [25,26], and models project that by the year 2100 globally averaged seawater dissolved CO_2_ concentration will double from current levels to 750 ppm [27,28]. This increase is expected to result in a 0.4 decrease in globally-averaged surface water pH [25]. Previous research on the effects of elevated environmental CO_2_ on respiration and acid-base balance in marine invertebrates [29–33] has provided useful information, but it is difficult to extrapolate from these studies to other marine invertebrates for two reasons: (1) not all organisms studied inhabit environments with natural variation in CO_2_; and (2) these studies examined only acute and short-term responses instead of responses over a complete life cycle as may be relevant to the time scale of anthropogenic CO_2_ increases. Recently, longer experiments have examined responses of invertebrates, in terms of physiological processes and performance, under elevated CO_2_ conditions [34–36]. These studies found significant effects in marine organisms that do not normally encounter high levels of CO_2_ in the natural environment. Long et al. [37] found that when female adult red king crab (*Paralithodes camtschaticus*) were exposed to elevated *p*CO_2_, the embryos were larger with smaller yolks, the larval hatching period was longer, and that larval survival decreased. For Tanner crab (*Chionoecetes bairdi*), survival decreased, growth slowed, and calcification was lower in crabs exposed to elevated *p*CO_2_, but morphology and condition indices were not affected [38].

A few studies have investigated the immune function of marine organisms exposed to elevated *p*CO_2_. When considering the immune response of marine organisms to ocean acidification, short-term (days to weeks) and long-term (months to years) effects may be different. Short-term experiments have been done on crabs (*Chionoecetes tanneri* and *Cancer magister*) [30] and the blue mussel (*Mytilus edulis*) [39,40]. Long-term experiments have been done on the blue mussel [41], the green sea urchin (*Strongylocentrotus droebachiensis*)[36], and the Norway lobster (*Nephrops norvegicus*) [42]. Short-term experiments tend to examine if and how quickly an organism is able to regulate physiology when challenged with ocean acidification; whereas, long-term experiments tend to provide more information about the general health status of an organism living with ocean acidification conditions. An example of a short-term experiment (7 days) was with the clam *Chamelea gallina* and the mussel *Mytilus galloprovincialis* exposed to varying pH and temperature at three different salinity levels [43]. Results indicate that ocean acidification with increased temperature resulted in significant changes in total hemocyte counts, Neutral red uptake, lysozyme activity and total protein levels for both bivalve species between treatments. Longer term studies (1–6 months) have been done on the Norway lobster, *N*. *norvegicus* [42], blue mussel, *M*. *edulis* [40,41], and the sea star *Asteria rubens* [44]. All long term studies found that elevated *p*CO_2_ had an effect upon the immune functions of these marine invertebrates. This research suggests that understanding the effects of elevated *p*CO_2_ upon the innate immune functions of marine invertebrates is necessary to assess how ocean acidification may modify hemocyte response under different environmental conditions.

In the present study, we sought to determine if there was a difference in hemocyte immune characteristics of Tanner crab, *Chionoecetes bairdi* a Brachyuran species, after being exposed to long term (2 years) acidified ocean water. Tanner crabs are a commercially and ecologically important species that live in northern latitude waters of the Pacific Ocean where ocean acidification is expected to occur to a greater extent and faster than in tropical waters because of water temperatures and changes in ocean circulation [45]. Ovigerous Tanner crabs were reared in ambient pH, pH 7.8 and pH 7.5 waters for two years. This study is a component of a larger project that also examined embryo development, mean number of viable larvae hatched, hatching success, mean hatch duration, mean brooding duration, larval survival, larval morphology, larval calcification, and calcification of the adult crabs under elevated *p*CO_2_ [46]. In the present study, the null hypothesis was that Tanner crabs exposed to more acidic water for two consecutive years would not differ from control crabs in hemolymph pH (pH_e_) and several hemocyte physiological variables: total counts, internal pH (pH_*i*_), viability, and phagocytosis. These hemocyte variables then were plotted against total number of eggs in a clutch, viable eggs/non-viable eggs, and the calcium content in the shell of the adults from the same experimental crab [46] to determine if a possible relationship could be observed.

## Methods

### Crab Exposure Treatments

Currently, ethical approvals for research on invertebrates are not required by federal, state, or international law. Field collection and transportation of Tanner crabs was authorized by Alaska Department of Fish and Game Fish Resource permits (CF-11-028; CF-12-022). In May and June 2011, ovigerous Tanner crab with un-eyed embryos were collected with pots and a small otter trawl from Chiniak Bay, near Kodiak Island, and transferred to the Alaska Fisheries Science Center Kodiak Laboratory seawater facility in Kodiak, Alaska.

Water was acidified using the same methods as Long et al. [37]. Incoming filtered seawater was bubbled with CO_2_ to a pH of 5.5. In two head tanks, ambient sea water was mixed with the acidified seawater to pH 7.8 or 7.5 (expected pHs in ~2100 and 2200). A Durafet III pH probe in each of the head tanks was used to control the flow of pH 5.5 water into the head tanks to regulate the pH. A third head tank (ambient) did not receive any pH 5.5 water. Individual experimental tubs (68 L) with Tanner crab were then supplied with water from one of the three head tanks at 1L min^-1^. Daily measurements of pH and temperature were taken using a Durafet III pH probe. The pH was kept within 0.02 pH units of the target value.

Water samples were taken from each treatment tank weekly to measure pH, total alkalinity (TA), and dissolved inorganic carbon (DIC). The pH, alkalinity, and DIC values of each container were used to back-calculate the saturation state of calcite (Ω_ca_, prevalent calcium-carbonate crystal in the crab) and the *p*CO_2_ of each treatment using R SEACARB [47]. Measurements for pH were determined spectrophotometrically following the protocol outlined in the Guide to Best Practices for Ocean CO_2_ Measurements [48]. The TA and DIC measurements were sent out for analysis to the University of Alaska in Fairbanks and NOAA/AFSC lab (Juneau, AK) where Certified Reference Material from the Dickson Laboratory (Scripps Institute, San Diego, CA) was used to assure accuracy of alkalinity and DIC measurements. For further details, see Foy et al. [46]. Table 1 shows the measured pH, temperature, DIC, TA, and calculated Ω_ca_ and *p*CO_2_ values. The average pH (± standard deviation, SD) for each of the treatments was 7.50 ± 0.03, 7.80 ± 0.03, and ambient (8.09 ± 0.07). The average salinity for the experiment was 31.2 ± 0.3. The experiment ended 15 July 2013 after crabs had been held under elevated *p*CO2 for 2 years.

**Table 1:** Average carbonate chemistry with standard deviation in brackets from July 2011 to July 2015 for the long-term ocean acidification experiment with Tanner Crab (*Chionoecetes bairdi)*. *p*CO_2,_ partial pressure of carbon dioxide in μatm; HCO_3_^-^, bicarbonate ion in μmol kg^-1^; CO_3_^-2^. bicarbonate ion in μmol kg^-1^; DIC, dissolved inorganic carbon in μmol kg^-1^;ALK, alkalinity in μmol kg^-1^; Ω_Ar_, calculated aragonite saturation state; Ω_Ca_, calculated calcite saturation state.

Treatment	pH	*p*CO_2_	HCO_3_^-^	CO_3_^-2^	DIC	ALK	Ω_Ar_	Ω_Ca_
**Ambient**	8.09 (0.07)	391.90 (65.59)	1.90 (0.04)	0.09 (0.02)	2.01 (0.04)	2.13 (0.06)	1.44 (0.25)	2.31 (0.40)
**pH 7.8**	7.80 (0.03)	781.17 (31.13)	1.99 (0.04)	0.05 (0.00)	2.08 (0.04)	2.13 (0.06)	0.78 (0.05)	1.24 (0.07)
**pH 7.5**	7.50 (0.03)	1597.15 (62.76)	2.05 (0.04)	0.03 (0.00)	2.16 (0.04)	2.13 (0.04)	0.40 (0.02)	0.64 (0.04)

At the beginning of the experiment, there were 48 crabs with 16 in each treatment. Crabs were checked daily for mortality and fed frozen fish and squid to satiation twice per week throughout the experiment. Water was chilled when necessary and allowed to fluctuate seasonally to ensure appropriate temperatures. Over the course of the experiment, the highest survival rate was in the ambient treatment (63%), followed by 7.5 pH treatment (44%), and the lowest in the 7.8 pH treatment (38%); however there was no significant difference between treatments [46]. Thus, by 15 July 2013 there were only 23 crabs left for the hemocyte analysis with n = 10 in ambient conditions, n = 6 for the pH 7.80 treatment, and n = 7 for the treatment of 7.50. Plots were generated to determine if a relationship of the data from this study and from the total number of eggs in the second clutch, viable/non-viable eggs, and calcium concentration in the adult shell to determine if there were relationships between these variables.

Other researchers have found that immune function at ambient conditions for final sampling (t_f_) was significantly different than initial hemocyte function, t_0,_ which was attributed to stress from laboratory conditions (i.e., handling, the artificially constructed mesocosm) [40,41]. For practical and methodological reasons, all immune function variables in crabs could only be measured at t_f_, as Hernroth et al. [42] did for the Norway lobster. After 2 years of exposure, hemolymph was taken from each crab to examine hemocytes. The females ranged in carapace width 89–112 mm, with an average of 99 ± 1 mm (Standard error, S.E.). There are many variables that can be measured to assess immune function, including viability, phagocytosis, pH (pH_e_ and pH_i_), adhesion, and respiratory burst. We originally took samples for all of those immune functions; however, during analysis of t_f_, the flow cytometer malfunctioned. As a consequence, data for viability, phagocytosis, and pH were the only variables analyzed before the crabs were sacrificed for other measurements [46].

### Crab Hemolymph Collection

**Fig 1:**
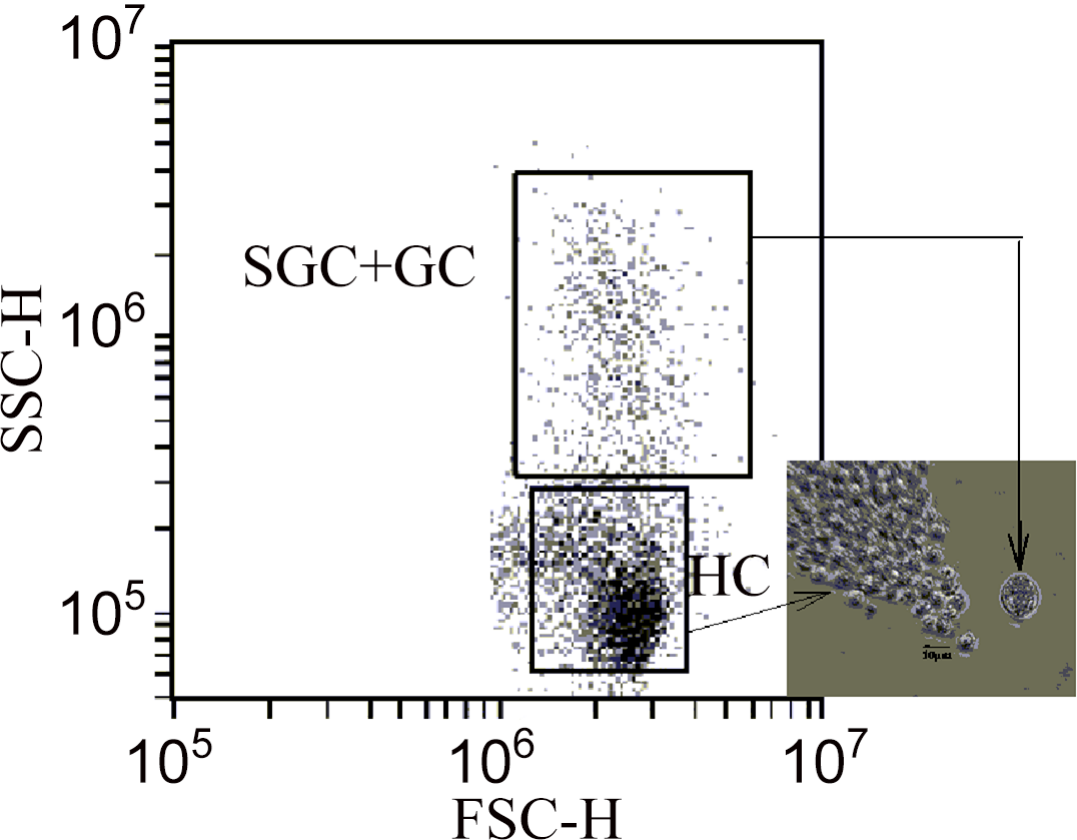
Forward Scatter (FSC-H) versus Side Scatter (SSC-H) of hemolymph from *Chionoecetes bairdi*, Tanner Crab.

A 2-ml, 23-gauge, sterile needle was used to collect hemolymph from each crab. The needle was inserted in the membrane of the back walking leg adjacent to the carapace. In certain crabs, proteins found in hemolymph can cause coagulation of hemolymph within minutes, thus anticoagulant solutions may need to be used during hemocyte assays. The use of anticoagulant can be problematic because the anticoagulant can cause cell lysis thus effecting immunological assays [17]. Using the flow cytometer, we tested the need to use an anticoagulant with Tanner crab hemolymph by bleeding a crab, then quantifying the number of cells counted by the flow cytometer over a 4 hour time period (the longest immunological test). There appeared to be little coagulation occurring for Tanner crab hemolymph; therefore, an anticoagulant was not used for any hemocyte measurements. There were only two hemocyte populations that could be distinguished easily on the flow cytometer, based upon internal complexity and forward-angle light scatter (Fig 1). The flow cytometer could not distinguish SGC from GC categories; therefore, data for HC and combined SGC+GC were reported.

### Hemocyte Analysis Protocols

All hemocyte measurements were made on individual Tanner crabs (no pooling of hemolymph) and were run on an Accuri C6 flow cytometer (Becton Dickinson, United States) for 30 seconds at 66 μl min^-1^. Side-scatter, forward-scatter, and fluorescence intensities of individual cells were collected and analyzed using BD Accuri C6 Software (Becton-Dickinson, United States). Following the guide of common practices for flow cytometer, sheath fluid for the flow-cytometer was 0.22 -μm-filtered local seawater [49]. Flow-cytometric hemocyte analysis protocols, which are commonly used in oysters, for viability and phagocytosis were adapted from Hégaret et al. [50]. As flow-cytometric hemocyte protocols have not been applied previously to Tanner crab, a thorough description of each measurement is presented below.

#### Viability

Hemocyte samples were diluted for use in the flow cytometer so that final cell counts were between 10^5^ to 10^6^ cell ml^-1^. Hemolymph dilution was with 0.22-μm-filtered sheath fluid (local seawater), which is common in flow cytometry [49]. To determine the number of viable hemocytes, a 150-μl subsample of hemolymph was mixed with 150 μl of sheath fluid, with 4 μl of 100,000x diluted SYBR Green® (S-7585, Life Technologies) and 20 μg ml^-1^ of propidium iodide (P4170, Sigma). SYBR Green® is a stain that crosses cell membranes and attaches to nucleic acids in both living and dead cells. Once attached it has a fluorescence emission at 520 nm, which is detected in the FL1 detector of the Accuri C6 (533 ± 30 nm). Propidium iodide, on the other hand, cannot cross the membrane of viable cells, but will attach to DNA within dead cells and has a fluorescence emission of 617 nm, making the cells detectable in the FL3 channel (610 ± 10 nm) of the flow cytometer. Falcon tubes containing hemolymph and reagents were incubated at 20°C for 1 hour in the dark before being analyzed on the flow cytometer. Counts of live/dead cells for HC and SGC+GC were determined.

#### Phagocytosis of fluorescent microbeads

A 150-μl subsample of hemolymph was mixed with 150 μl of 0.22-μm-filtered sheath fluid. A stock solution of 5.68 x 10^9^ particles ml^-1^ of fluorescent beads (Fluoresbrite YG Microspheres®, 18338, 2.00 μm; Polysciences) was added to each tube so that the final concentration of fluorescent beads in assay tubes was 1.01 x 10^7^ ml^-1^. Tubes were incubated in the dark for 2 hours at 20°C. The beads used were detected with the FL1 detector. It is difficult to discriminate hemocyte populations in this assay because phagocytosis of beads may cause changes in cell size and complexity [50]; therefore all hemocytes were grouped as one population. All hemocytes that contained 3 beads or more on the FL1 histogram were considered to be highly phagocytic [50].

#### pH

Following closely the protocols of other researchers who measured hemolymph pH (pH_e_) [42,44,51], we used conical-bottom centrifuge tubes that fit the pH probe. pH measurements were done immediately after extraction of the hemolymph (1–2 minutes). No drift caused by temperature change was observed in the 5 seconds needed to record pH_e_. Hemolymph pH (pH_e_) was determined using a Corning pH-30 electrode calibrated with NBS buffers.

Measuring the pH of the hemocytes (pH_i_) of living cells using fluorescent probes has been done in yeast cells [52], bacteria [53], and mammals [54]. All of these methods had a similar approach wherein a calibration curve first was established, and then the pH_*i*_ was calculated based upon the calibration curve. For pH_*i*_ the cell-permeable fluorescent probe Carboxy SNARF® (Life Technologies) has a pH-dependent wavelength shift in fluorescence from 580 nm to 640 as a solution becomes more basic, thus it can be utilized to determine pH_i_. The excitation wavelength of the probe is 480 nm allowing for FL2 and FL3 detection with the flow cytometer. The ratio of the FL2 and FL 3 is calculated and plotted against the measured pH of the solution. The best fit of the line was then utilized to calculate pH_i_ of the samples.

Carboxy SNARF® comes in a variety of forms that allow for the determination of pH_i_ in selected ranges from 6.0 to 9.0. Carboxy SNARF-5F® (S-23923, Life Technologies), with a pK_a_ value of 7.2, provides more robust measurements of pH_i_ in the range found within crab hemocytes. To be able to determine pH_i_, an *in situ* calibration is necessary. This is achieved by using the ionophore nigericin (N1495, Life Technologies) and potassium (K^+^) to equilibrate the intracellular pH with the extracellular environment [54]. The K^+^ buffer solution causes the membrane potential around the cells to become 0 mV, thereby equilibrating the H^+^ ion within the cells to the pH of the K^+^ buffer [55]. Accordingly, by varying the pH of the K^+^ solution (7.0–8.5) in the presence of nigericin, H^+^ ions will equilibrate within and outside of cells. To reiterate, the intracellular and extracellular hydrogen ion concentration will be equal to the intracellular-to-extracellular potassium ion ratio in the presence of nigericin [56,57]. Carboxy SNARF-5F® had not been used with crustaceans, but has been used in marine organisms including foraminifera, corals, and phytoplankton [52–54,58–60]. It is important to ensure that the K^+^ buffer solution does not deform the cells while still allowing intracellular H+ to equilibrate with the K^+^ buffers. Life Technologies, SNARF probe manufacturer, recommends starting with K+ buffers that range from 100 mM to 150 mM K^+^, which is usually the range of K^+^ in the cytosol. There is little known to date of the K^+^ in crustacean hemocytes; therefore, an experiment testing different K^+^ levels (100 to 150 mM in 10 mM intervals) and different nigericin levels (5–15 μM nigericin dissolved in 200 proof ethanol in 5 μM intervals) was performed. The concentrations of K^+^ and nigericin listed above are the final concentrations in the staining tube with all added reagents and hemolymph. Two methods were applied to determine if the hemocytes were affected by the K^+^ and nigericin solution (1) flow cytometry analysis and (2) visual confirmation under the microscope. Ruptured hemocyte cells in the K^+^ buffer with nigericin and the SNARF produced different forward and side scatter plots (FSC-H and SSC-H, respectively) compared to hemocyte cells in seawater. Before analysis with the K^+^ buffer, the mean FSC-H and SSC-H for both SCG+GC and HC cells were determined for 5 Tanner crabs. The variability in FSC-H and SSC-H between these individuals averaged 6.1 ±1.0% for HC and SGC+GC cells. Table 2 shows mean FSC-H and SSC-H at each K^+^ buffer and nigericin combination relative to an untreated Tanner crab hemocytes. The test tube that had a final stock solution of 140 mM of K^+^ buffer and 5 μM nigericin had the lowest % change relative to the control sample (<5%). Therefore, after analyzing the matrix of different K^+^ and different nigericin levels, the following K^+^ stock solution was employed: 280 mM potassium chloride, 2 mM magnesium chloride, 4 mM calcium chloride, 10 mM glucose, 20 mM TRIS, and 20 mM HEPES. All of the above listed chemicals for the K^+^ solution were analytical research grade. This K^+^ solution was similar to those used in other SNARF-5F® research [51–53]. The K^+^ buffer was titrated between the range of 7.4–8.5, based upon research by others that suggested that pH_i_ of invertebrates can range from 7.4 to as high as 8.1 [61–63]. To 150 μl of one of the known pH-adjusted K^+^ solutions, a 150 μl subsample of hemolymph was added. This yielded a final K^+^ buffer solution of 140 mM potassium chloride, 1 mM magnesium chloride, 2 mM calcium chloride, 5 mM glucose, 10 mM TRIS, and 10 mM HEPES. Nigericin is highly unstable in aqueous solution and adheres to plastics, so it was added directly to the calibration buffer to give a final concentration of 5 μM in the tube. After the addition of nigericin, 4 μL of the working stock solution 300μM of SNARF-5F® was added to the tube (final concentration in tube 2 μM). The SNARF-5F® working stock solution was prepared by diluting with anhydrous dimethyl sulfoxide (DMSO). The final osmolality was estimated at 650 mOsmol Kg^-1^, which was on the lower end of invertebrate osmolality(300–1,400 [42,64–66]); however, we found little difference in the FSC plots and SSC plots of cells (Table 2) and no visual damage under the microscope using this osmolality solution compared to non-treated cells.

**Table 2:** The percent change of the mean FSC-H and SSC-H for both hyalinocyte cells (HC) and semi-granular+granular cells (SGC+GC) relative to a sample of hemocytes without the potassium buffer, nigericin, or SNARF from Tanner crab (*Chionoecetes bairdi)*. The potassium and nigericin concentrations listed are the final concentrations in the test tube after all the reagents and hemolymph were added.

Potassium (mM)	Nigericin (μM)	% HC FSC-H	%HC SSC-H	% SGC+GC FSC-H	% SGC+GC SSC-H
100	5	-14.0	8.9	2.8	8.3
100	10	-16.0	14.8	13.1	10.8
100	15	-1.6	11.0	19.3	11.1
120	5	-16.1	12.2	-0.4	11.4
120	10	-12.0	14.8	13.2	12.6
120	15	-0.3	-1.0	19.0	8.0
140	5	1.7	1.6	4.9	4.0
140	10	-7.2	9.5	12.1	17.1
140	15	-8.0	4.8	16.5	9.5
150	5	-7.0	2.6	2.6	7.1
150	10	-7.4	14.8	14.5	14.1
150	15	+10.2	1.5	25.2	2.7

**Fig 2:**
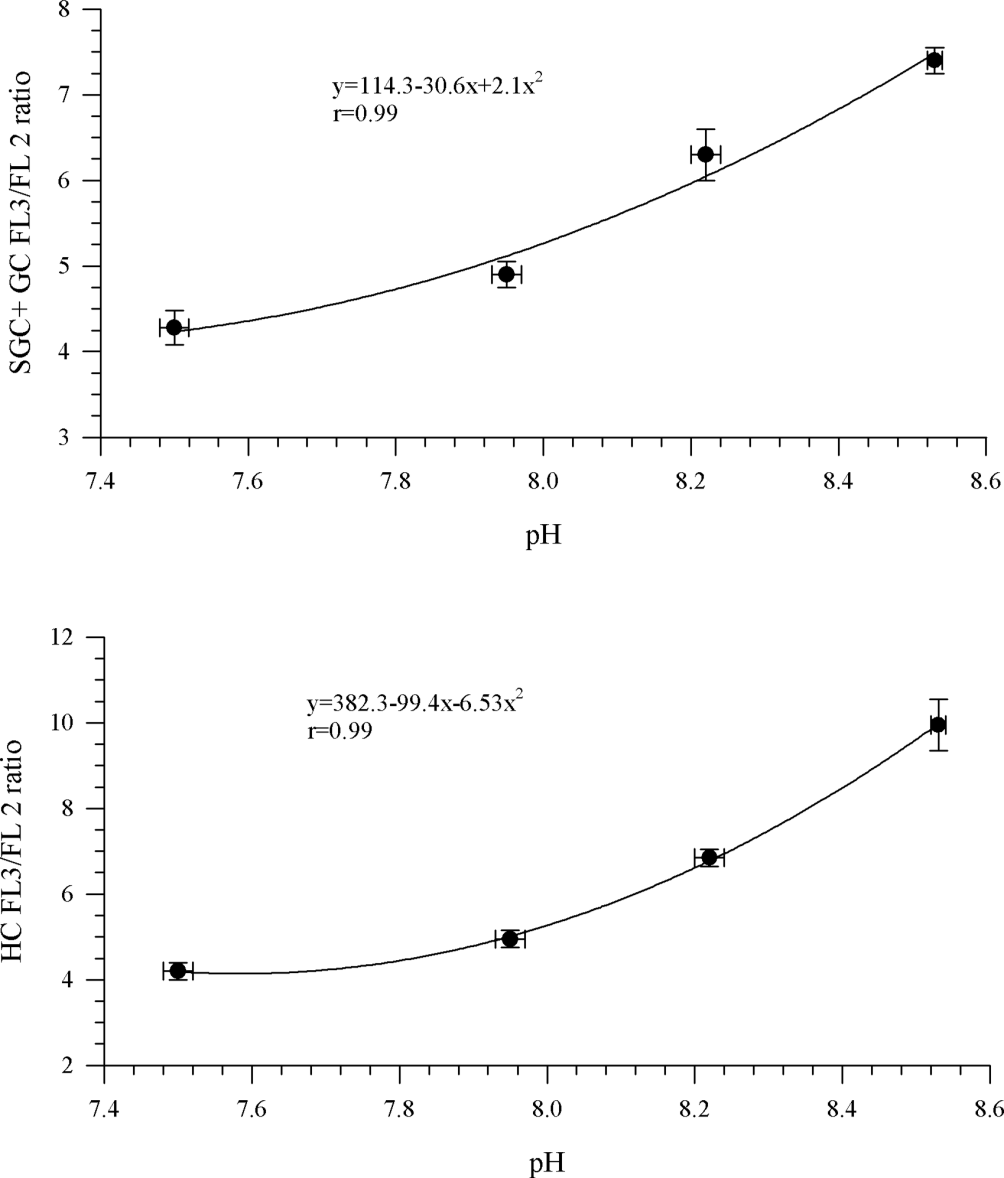
The calibration profile of Carboxy SNARF® for *Chionoecetes bairdi*, Tanner Crab. The slope of the line was used to calculate pH_i_ of semi-granular+ granular cells and hyalinocyte cells.

Ideally, if enough hemolymph is available, separate calibration curves for 5 individual crabs should be generated to examine variance; however, this can be limited by the amount of hemolymph available. Thus, for the calibration curve we randomly chose 5 individual crabs from which 150 μl of hemolymph was taken and pooled to yield a final, total, pooled volume of 900 μl hemolymph. For the pool hemolymph 4 tubes were prepared with 150 μl of known K^+^ buffer solutions + 150 μl of pooled hemolymph + 4 μl of stock nigericin + 4 μl of stock SNARF-5F®,(the calibration curve samples). The samples for the calibration curve then were allowed to react for 30 minutes at 20°C in the dark before reading on the flow cytometer. The probe SNARF-5F® has a characteristic pH, so to reduce errors before analysis on the flow cytometer, pH of the buffered samples was measured with the Corning pH-30 electrode, and this value was the measured pH in the calibration curve (Fig 2.) The FL3/FL2 ratios of hemocytes in the calibration curve samples were used to generate a calibration curve that was used to extrapolate pH_i_ for the hemocytes. The calibration curve does have an error associated with it of 0.1 pH units (Fig 2).

To determine the pH_i_ of the HC and SGC+GC from each crab, a 150-μl subsample of hemolymph was mixed with 150 μl of 0.22-μm-filtered sheath fluid. A stock solution of 375 μM of SNARF-5F® was added to each tube so that the final concentration of SNARF-5F® was 5 μM (the same amount used in the calibration curve). The samples were allowed to react for 30 min at 20°C in the dark before reading on the flow cytometer. Using the calibration curve, the FL3/FL2 ratio could be used to calculate pH_i_ of HC and SGC+GC. Unfortunately, after analysis it was determined that most of the HC values had a calculated pH_i_ value below our lowest standard of pH 7.4 (Fig 2). When this problem became apparent, we could not go back and remake another calibration curve using the hemolymph from the experiment (instrument had broken during analysis). We knew that as the probe approaches its pK_a_ value (7.2) it is designed to produce a strong quadratic relationship; therefore, using other adult Tanner crabs, not from this experiment (after the instrument was fixed), we ran multiple calibration curves (n = 10). This time we had a potassium buffer solution at a pH of 6.7. We found that, if the quadratic equation was well established, using a calibration curve with a pH value at 6.7 versus 7.4 resulted in a small calculated pH_i_ difference (±0.04, n = 10 curves). With the quadratic relationship already established in our standard curve (Fig 2), we were reasonably confident that we could estimate the pH_i_ of the HC cells accurately.

### Adult Parameters

The total number of eggs in the second clutch, percent viable eggs, and percent non-viable eggs were estimated for each female. Prior to larval hatching, nets were placed on the outflow of each tub to retain all of the larvae from each female and newly hatched larvae were collected and dried daily at 60°C until a constant weight was achieved. Average weight per larva for each female was calculated from 3 to 6 replicates (number varied with female) of 50 dried larvae. The average weight per larva for each female was used to calculate how many larvae each female hatched daily. If less than approximately 200 larvae hatched, then all of the larvae were counted. For each female, total number of viable larvae hatched was calculated by adding together the number of larvae hatched daily. For each female, the debris from pleopod cleaning was collected, examined microscopically, and all viable larvae, non-viable larvae, and dead eggs counted in a volumetric subsample. Non-viable larvae that hatched were larvae that did not molt past the pre-zoea stage to the first zoeal stage. Percent viable eggs was defined as the percent viable larvae hatched divided by the calculated total number of eggs that could have hatched (number of viable larvae hatched + number of non-viable larvae hatched + number of eggs that did not hatch). The percentage of non-viable eggs (non-viable larvae hatched + number of eggs that did not hatch divided by the total number of eggs that could have hatched) was also calculated. For further details, see Foy et al. [46].

At the end of the experiment (July 2013), calcium content was analyzed for 9 ambient, 5 pH 7.8 and 5 pH 7.5 treatment females from the same portion of their carapace (approximately a square centimeter of the posterior margin region) by Gel laboratory (Charleston, SC).

### Statistical Analysis

All measured variables were analyzed with a one-way analysis of variance with pH-exposure treatments as the factor. Each variable was tested for normality using the Shapiro-Wilkes test and for homogeneity of variance using Levine’s test. The number of cells live and dead was log_10_ transformed and percentage data were arc sin of the square root transformed to meet the assumptions of ANOVA. For the ANOVA the P value, the degrees of freedom (df), and the F-ratio were reported for all analyses. For each analysis in which there was a significant difference between treatments, the average ± standard error (SE) also was reported.

There was not sufficient range overlap among treatments for an ANOVA with a co-variant to statistically relate hemocyte variables with other variables measured in the same crabs. Therefore for the hemocyte variable were there was a significant difference between treatments a plot against Ca concentration in adult shell, total number of eggs in the second clutch, and % of viable/non-viable eggs was done to determine if a there was a significant correlation (p<0.05). It is noted that there is some interdependence between the treatments. All statistical analyses were conducted using Statgraphics Plus statistical software (Manugistics, Rockville, MD, USA).

## Results

**Fig 3:**
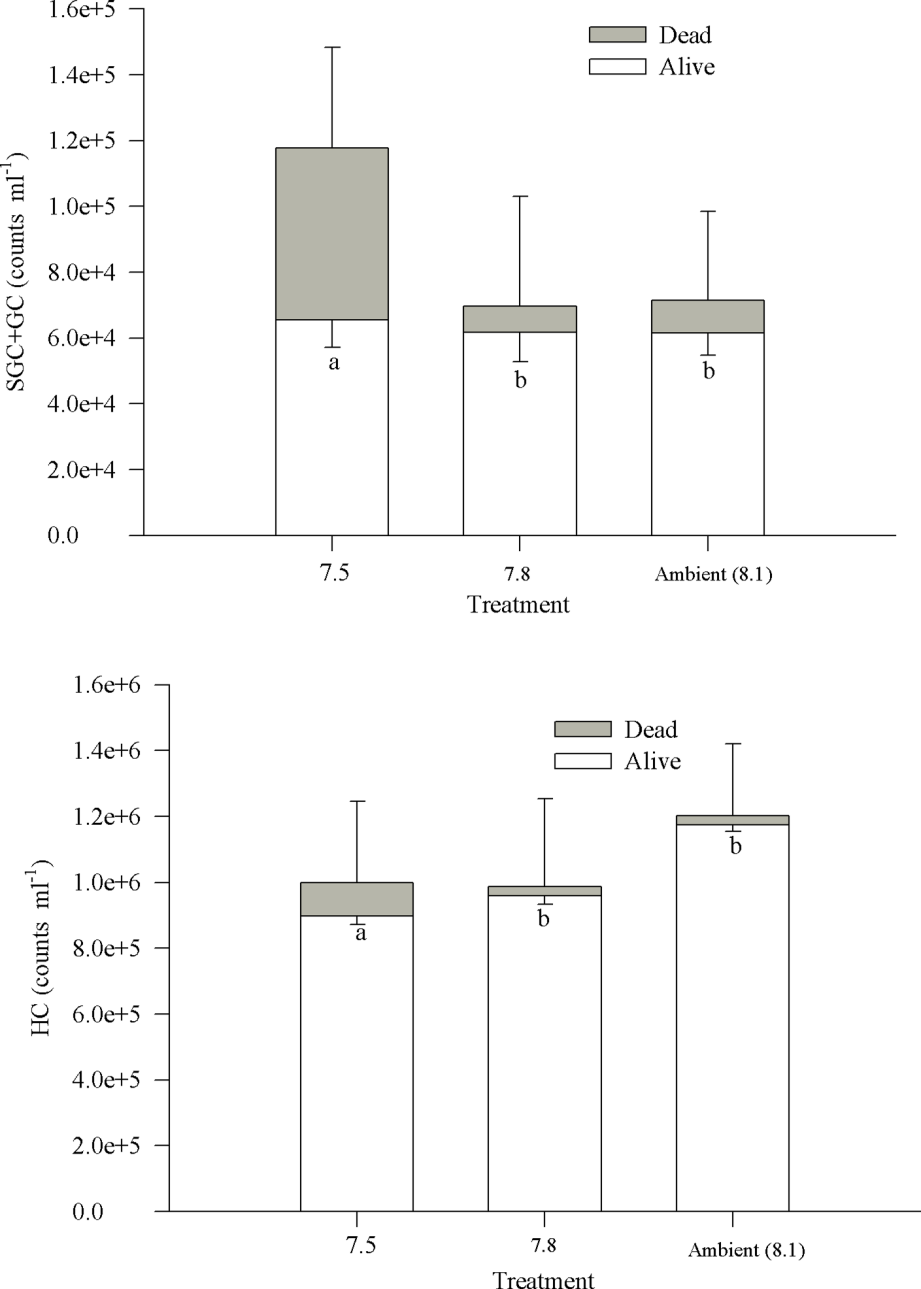
Live and dead semi-granular+granular cells (SGC+GC) and hyalinocyte cells (HC) for *Chionoecetes bairdi*, Tanner Crab. The upper region contains the semi-granular and granular cells, while the lower region contains the hyalinocytes. The error bars for the dead cells are negative, while the error bar for the live cells is positive. Different homogenous groups are labeled for only the dead cells since there was no significant difference in the number of total cells.

The total number of hemocytes suspended in the hemolymph of Tanner crabs (SGC+GC and HC) averaged 1.2 ±0.2 x 10^6^ cells ml^-1^ (standard error, SE), with no significant difference (P = 0.39,df = 22, f-ratio = 0.67) between treatments. For the types of hemocytes, the SGC +GC were a smaller percentage of the total cells then the HC (Fig 3). The percentage of total live cells ranged from 79% to 99% of the population, and there was no significant difference between treatments (P = 0.12, df = 22, F-ratio = 1.23); however, the percentage of total dead cells in circulation ranged from 1% to 31%, and there were significant differences between treatments (P<0.01, df = 22, F-ratio = 6.05). The number of dead cells was significantly higher at the pH treatment of 7.50 than in the pH 7.80 and ambient treatments (Fig 3). To determine if there were more dead SGC+GC or HC, the percentages of live and dead cells for each population were determined (Fig 3). There was a significant difference in the number of dead SGC+GC between treatments (P<0.01, df = 22, F-ratio = 9.95), with no difference between the ambient and 7.80 pH treatments and the highest percentage of dead cells in the 7.50 pH treatment. The HC had a lower percentage of dead cells than the SGC+GC category. As with the SGC+GC, there were significantly more dead HC in crabs held at the lowest pH 7.50 compared to the other two treatments (P<0.01, df = 22, F-ratio = 9.95).

**Fig 4:**
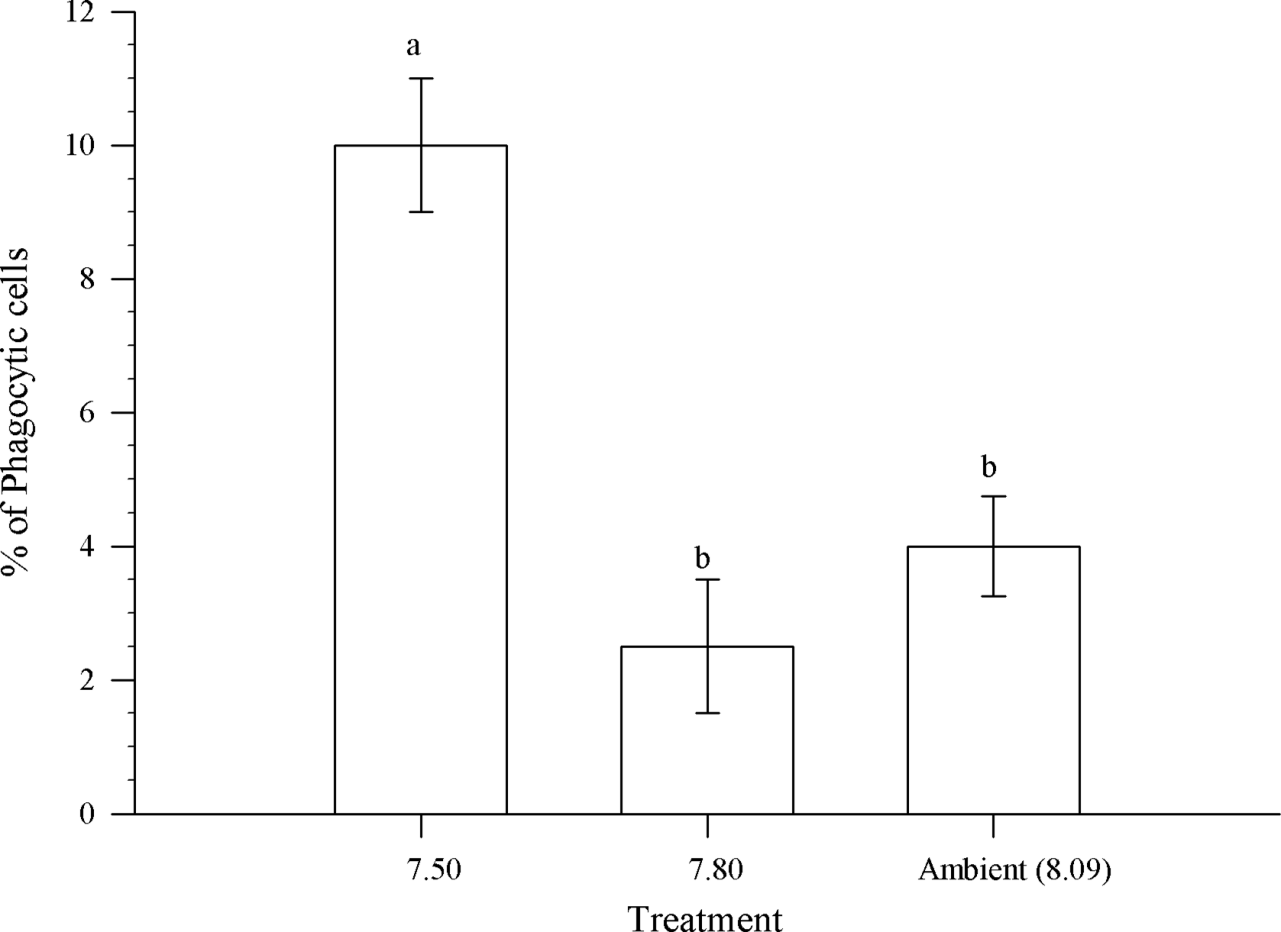
The percentage of phagocytic hemocytes in *Chionoecetes bairdi*, Tanner Crab, from each pH treatment. The error bars represent the standard error and a different letter indicates a significant difference between treatments.

There were significant differences between treatments in the percentage of highly-phagocytic hemocytes (P<0.01, df = 22, F-ratio = 4.99; Fig 4). Crabs held in pH 7.80 had the smallest percentage of phagocytic cells; whereas, the 7.50 treatment had the highest number. Crabs held in the ambient treatment were statistically indistinguishable from the 7.80 treatment for number of phagocytic cells.

**Fig 5:**
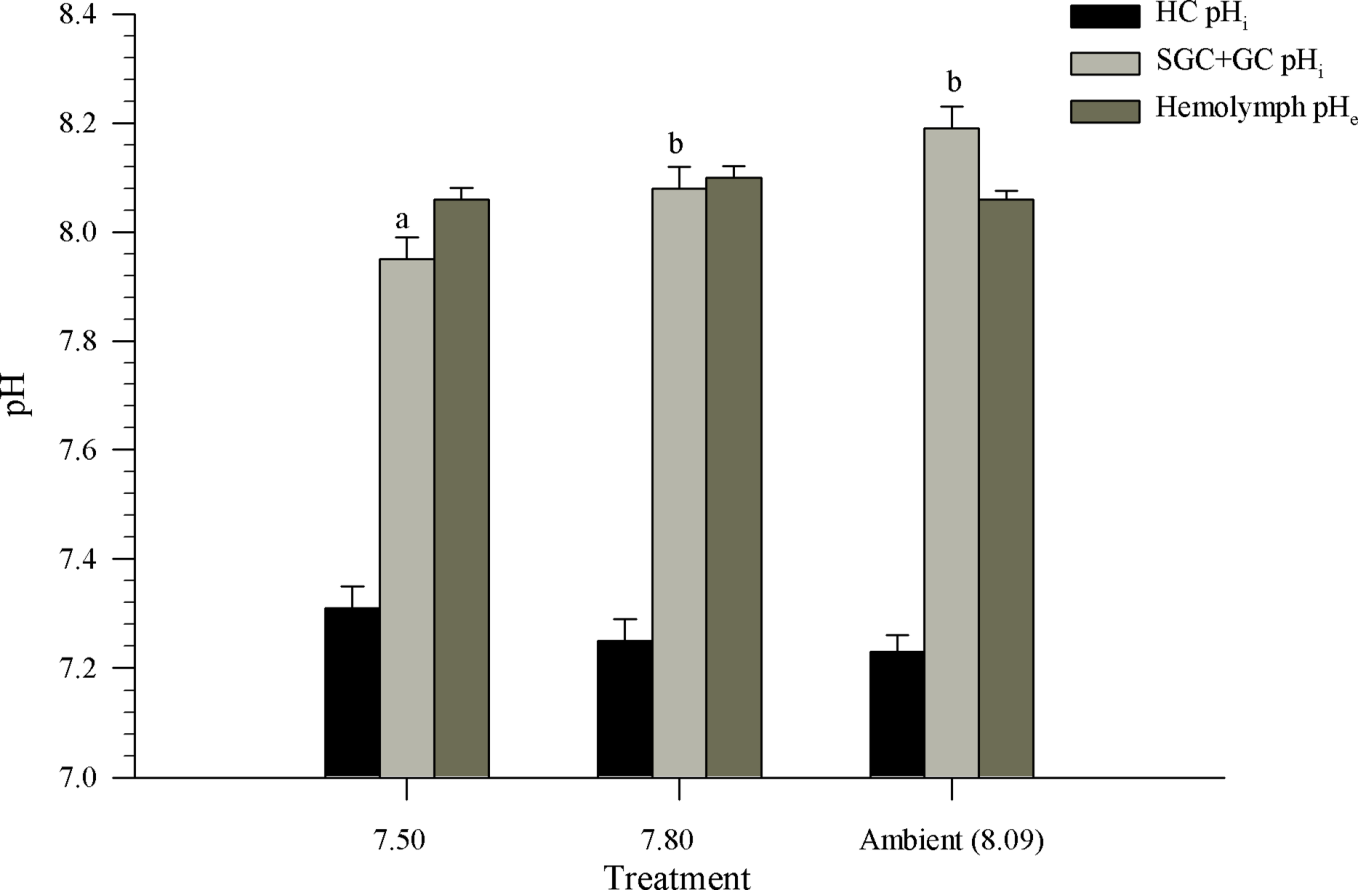
Hemolymph pH (pH_e_) and intracellular pH (pH_i_) for semi-granular+granular cells (SGC+GC) and hyalinocyte cells (HC) from *Chionoecetes bairdi*, Tanner crab. The error bars represent the standard error and letters indicate significant differences between treatments.

The pH_e_ of the hemolymph ranged 8.01–8.22. The mean pH_e_ of the hemolymph was 8.09 ± 0.05 and did not differ significantly between treatments (P = 0.21, df = 21, F-ratio = 1.69; Fig 5). Unlike the pH_e_, the pH_i_ of the SGC+GC ranged from 7.83 to 8.32 and varied significantly between treatments (P<0.01, df = 21, F-ratio = 7.61). The pH_i_ of the SGC+GC for the ambient and pH 7.8 treatments were higher (8.08 ± 0.05) than the pH_i_ of hemocytes in crabs held at 7.50 (7.95 ± 0.02; Fig 5). The 7.80 treatment SGC+GC had a pH_i_ similar to the ambient treatment (8.19± 0.06). Unlike the SGC+GC, the mean, estimated pH_i_ of the HC was not significantly different among treatments (P = 0.29, df = 21, F-ratio = 1.33). The pH_i_ of the HC ranged from 7.13 to 7.52 (Fig 5), with an overall mean of 7.24 ± 0.03. The HC had a lower pH_i_ than the SGC+GC.

**Fig 6:**
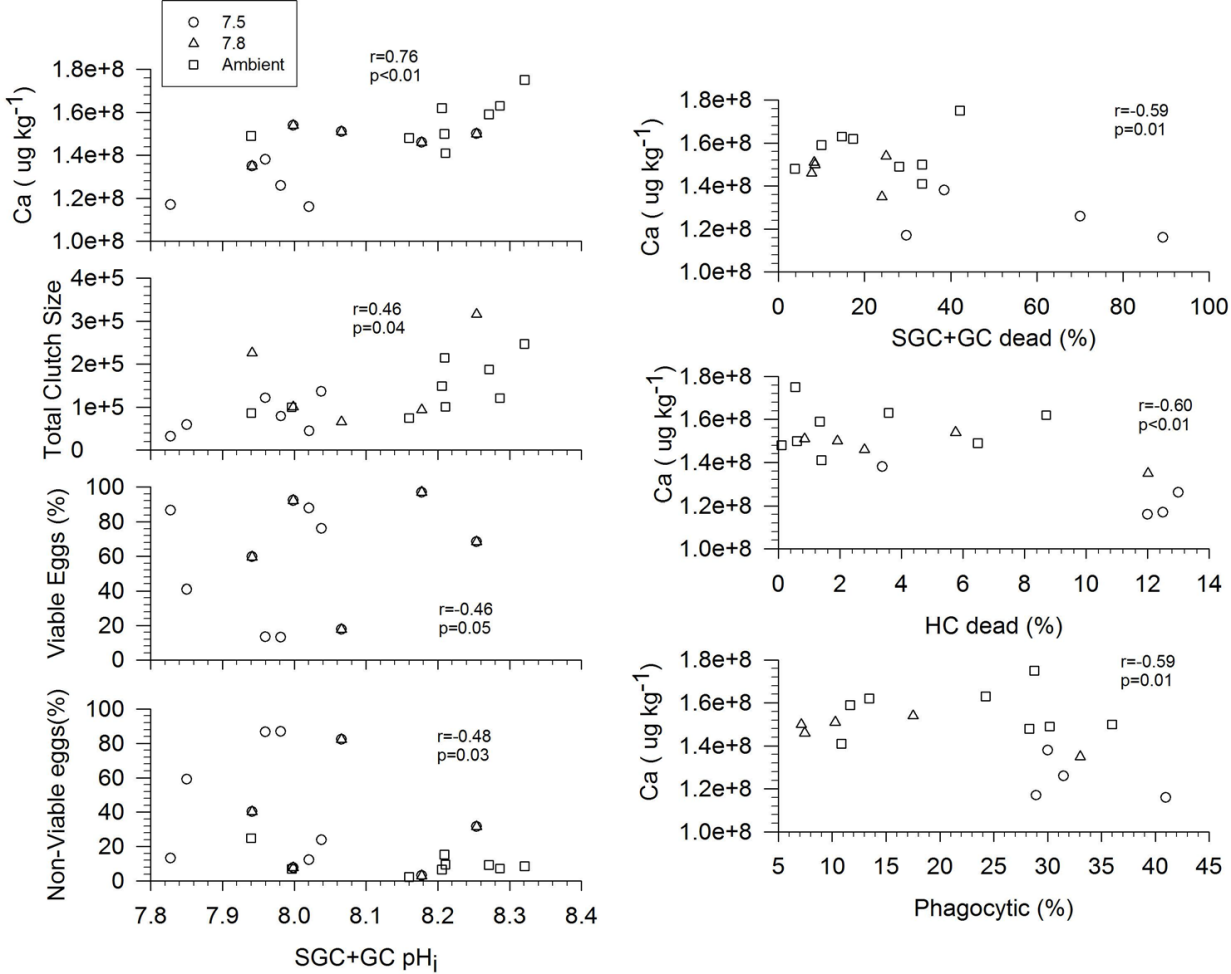
Relationship between hemocyte function in and parameters measured in the adult *Chionoecetes bairdi*, Tanner Crab. The hemocyte function that showed an relationship include SGC+GC pH_i_, phagocytosis, and % dead hemocytes relative to the adult parameters for number of eggs in second hatch, % viable/non-viable eggs, and calcium concentration.

There appeared to be a significant linear correlation between hemocyte variables and total number of eggs, the % viable/non-viable eggs, and calcium content in the adults (Fig 6). A potential connection between calcium concentrations in the adults appeared to exist when plotted against a number of hemocyte variables (pH_i_, phagocytosis, and viability). For phagocytosis and % dead cells, there appeared to be a negative correlation with Ca concentrations. The only variable that had a positive correlation with Ca concentration was SGC+GC pH_i_. The total number of larvae hatched in the second hatch, and the viability of the eggs had a positive correlation with the pH_i_ of both SGC+GC. As the SGC+GC pH_i_ increased, the number of eggs and the % viable eggs increased.

## Discussion

Melzner et al. [67] predicted that highly mobile organisms, such as fish, cephalopods, and some crustaceans that are capable of controlling pH_e_ through active ion transport would be more tolerant of ocean acidification than organisms without strong ion-regulating mechanisms. Pane and Barry [30] reported that *C*. *tanneri* (another Tanner crab species) exhibited an extracellular, compensatory response to extreme external acidification (7.08) and was able to only slightly recover pH_e_ after 24 hrs exposure to acidified water. These authors hypothesized that following a longer exposure time to acidified water, *C*. *tanneri* might be able to recover pH_e_, as we observed with *C*. *bairdi*. This study revealed that, after 2 years of exposure to acidic water, Tanner crabs, *C*. *bairdi*, were able to regulate their pH_e_ in all treatments (8.09 ± 0.05). In short term experiments with various marine species of crabs, the reported pH_e_ ranged from 7.80–7.94 [30, 68, 69]. Weeks et al. [69] exposed *Carcinus maenas* to copper and found that within 7 days the pH_e_ decreased and only slightly recovered. Pane and Barry [30] had an initial pH_e_ of 7.8–7.9 and found pH_e_ dropped within 24 hours in *C*. *magister and C*. *tanneri* when exposed to acidic water. The pH_e_ reported here is slightly higher than other marine crustaceans, but the pH is not unusual for marine organisms. In *N*. *norvegicus*, the Norway lobster, after long term exposure to ocean acidification and elevated temperature, the control group had a pH_e_ of 8.3 [42]. Furthermore, in the marine benthic worm *Sipunculus nudus*, the control pH_e_ was reported to be 8.02 ± 0.03 [29]. Schründer et al. [70] found that Antarctic copepod pH_e_ ranged from 7.8–8.3. Even though our pH_e_ is slightly higher than some marine organisms, it is within the range found in the literature for crustaceans and other invertebrates. Although recovery time for *C*. *bairdi* in our experiments is unknown, it is likely that it is quicker than for *C*. *tanneri* given that habitat pH varies more for *C*. *bairdi*. A slow response to pH_e_ could lead to internal shell dissolution [71] and induce oxidative stress [72]. Further studies to determine the recovery time of hemolymph pH_e_ and intracellular pH_i_ for *Chionoecetes* spp. are warranted to further assess the potential for effects on internal shell formation.

Maintaining pH_e_ regulation is important to maintain oxygen supply to the tissues [73,74] and for acid-base equilibrium within the limits needed for protein/enzyme function; however, a range of pH_e_ has been noted in crustaceans under different environmental scenarios [30,42,68,69,75]. Unlike pH_e_, the pH_i_ is tightly controlled in most marine animals despite changes to the pH_e_ [75–77]. A crustaceans metabolism, protein synthesis, ion-regulation, and cell volume are effected by even small changes in the pH_i_ [75,78–80]. This study revealed differences in responses of SGC+GC and HC hemocytes in Tanner crab exposed long-term to lower pH levels. Hyalinocytes, thought to be responsible for phagocytic elimination of potentially pathogenic microbes [6,81], maintained a constant, mean pH_*i*_ of 7.24, independent of and lower than pH_e_. In marked contrast, in crabs held in ambient water, pH_*i*_ of SGC+GC hemocytes was higher than pH_e_ of the hemolymph. The pH_*i*_ of SGC+GC hemocytes in crabs from the lowest pH treatment appears to indicate an overall prolonged acidosis compared to crabs in the ambient treatment. Acidosis of pH_i_ has been observed in *S*. *nudus* [29] and the marine bivalve *M*. *edulis* [82], but often this acidosis recovers within hours to days after return to ambient conditions. It appears that the pH_i_ of HC cells is able to fully recover, while those of the SGC+GC cells were not. This fundamental difference in pH_i_ between hemocyte types is consistent with the current opinion that granular and agranular hemocytes in crustaceans originate from separate, stem-cell-like hematopoietic tissues [83] and underscore the importance of considering potential effects of ocean acidification upon crustacean hemocyte cell types separately, with further consideration of the different functions of each of the cell types.

Hemocytes are the main mediators of immunity in invertebrates, carrying out the phagocytic, encapsulating, and microbicidal responses that enable these animals to protect themselves against infection without a specific adaptive immune system [84,85]. Hemocyte cell have specialize functions, with division of function between types. The hemocytes also synthesize and store bioactive proteins that are released by exocytosis following interaction of pattern recognition receptors (PRRs) and non-self motifs carried on the surface of pathogens or parasites. SGC+GC hemocytes in crustaceans are thought to serve as hemolymph enzyme-regulating cells [83]. SGC+GC hemocytes were present in the hemolymph at 10^5^ cells ml^-1^ in the Tanner crabs analyzed. In addition, these cells participate in proPO, which has roles in both immune response and cuticle construction [83]. Arthropods and other invertebrates have the non-self reorganization system of proPO, which involves the copper enzyme phenoloxidase (PO). PO allows for the o-hydroxylation of monophenols and the oxidation of phenols to quinines [86]. After a cascade of several intermediate steps the proPO system results in melanization/sclerotization of isolated infective agents and of the cuticle during both wound repair and molting [83,86]. On first examination, by considering only the pH_i_ of the SGC+GC, it appears that these cells in Tanner crab are able to regulate pH_*i*_ independently of hemolymph pH_e_, with the individuals exposed to the most acidic ocean water regulating significantly less than the control. Regulation of pH_i_ would be consistent with the function that these cells must respond to subtle changes in hemolymph biochemistry.

One very noticeable effect of environmental acidification upon SGC+GC hemocytes in this study was the accumulation of dead cells in the hemolymph (Fig 3). This occurred only in the lowest pH treatment of 7.50 and was not associated with a reduction in the counts of live SGC+GC hemocytes. Death of hemocytes in any organism can be by apoptotic or “accidental” (sometimes referred to as ‘necrotic’) processes [87–89]. Although we were not able to obtain data on hemocyte apoptosis in this study, there was no visually apparent disease in any of the crabs that would cause necrotic death of hemocytes and other tissues. Assuming that the crabs were disease free, SGC+GC hemocytes likely were dying by apoptotic processes, then either apoptotic cells death was occurring at a faster rate in crabs held at pH 7.50, or apoptotic rates were similar in all pH treatments, and the processes that remove dead hemocytes were less effective at pH 7.50. Because phagocytosis of apoptotic, blebbing hemocytes is the main process of removing and recycling senescent cells in most organisms [90], we sought clues to the accumulation of dead SGC+GC hemocytes in data collected on the phagocytic hemocytes–the HC.

In the Tanner crab, HC were by far the most numerous cells in the hemolymph, with counts in the range of 10^6^ mL^-1^. Exposure of crabs to acidified water at pH 7.80 did not change the counts of live circulating HC. At the lowest pH tested of 7.50, counts of dead HC were significantly greater, but not extremely different, than in the higher pH treatments. Despite wide differences in *p*CO_2_ treatment, from 8.09 to 7.50, HC pH_*i*_ was regulated to a constant mean of 7.24 (Fig 5). Studies of tissue and intracellular pH in crustaceans and other arthropods are scarce, and those of HC and SGC+GC are nonexistent. Sartoris and Pörtner [61] reported a constant pH_*i*_ in shrimp (*Crangon crangon*) muscle of 7.4. Subsequently, Abele-Oeschger et al. [91] showed that pH_*i*_ in this shrimp species was lowered from a mean of 7.32 to 7.22 following five hours of exposure to elevated hydrogen peroxide to mimic oxidative stress. The Chinese mitten crab, *Eriocheir sinensis*, had a muscle-cell pH_*i*_ of 7.4 [63], and tail muscle pH_*i*_ in the prawn *Palaemon serratus* was 7.20 [92]. Thus, the constant 7.24 pH_*i*_ value found in Tanner crab HC is similar to pH_*i*_ reported in muscle cells of other crustacean species under natural conditions. Maintenance of pH_*i*_ in phagocytes is consistent with the need for the membrane to be relatively impermeable to small-molecular-weight molecules, such as reactive oxygen species and proteolytic enzymes that are released in phagosomes to kill microorganisms previously engulfed. The cell membrane that becomes the phagosome membrane must protect the cell from the extremely caustic environment within an active phagosome; therefore, general impermeability and very active ion pumps would be expected in the membranes of phagocytes. The relatively low pH within the phagocytes, compared to pH_e_, could be a consequence of respiratory carbon dioxide production [93] or simply a reflection of the general finding that physiological pH within the cytosol of most organisms tends to be just above neutrality [94].

Our findings indicated that the SGC+GC pH_*i*_ was clearly higher and different than that of the HC. But the reason why SGC +GC have a different pH_*i*_ then HC is not clear. A plausible explanation for the higher pH_*i*_ of SGC+GC could be attributable to the role that they play in crustacean cuticle formation and repair with the proPO system. Numerous studies of proPO pH have found that it can be as low as 6.0 and as high as 8.0, depending upon the species [95–101]. The higher pH range is thought to be associated with melanization/sclerotization of isolated infective agents and with the cuticle during both wound repair and molting of the carapace, but of the physiological reason for why the pH is higher under these conditions remains unanswered. High pH_i_ have also been found in organisms at sites of calcification. The tube worm, *Hydroides elegans*, had a pH_*i*_ that ranged from 8.5–9.0 [102]. In foraminifera, at the site of calcification the pH_i_ in the vesicles that containing the calcite varying from 7.5–9.0, with the large range most likely being an influence on cell age, calcification rate, and CO_2_ availability [58,103]. This increase in pH_i_ in crustaceans has also been observed during carapace formation during molting. In blue crab, *Callinectes sapidus*, the pH_i_ of the fluid in the carapace was 8.229 ±0.035 which was higher than that of the blood pH (pH_e_) 7.712 ± 0.017 [104]. Even though the pH_i_ is higher for SGC+GC cells, it is not uncommon in localized areas within a marine organism to have pH_i_ values as reported here, especially in cells that play a role in calcification. There are two models for the formation and repair of calcium carbonate in marine organisms: (1) the matrix model, and (2) the cellular model. The matrix model assumes that chitin proteins are the matrix structure, and acidic proteins control nucleation and calcium carbonate crystal growth; the cellular model assumes that cells mediate biomineralization, with granular cells playing an important role [105]. Hemocytes appear to have a special function in crustacean carapace formation and repair [106,107]. Histochemical visualizations have indicated that the granulocytes contain some of the proteins, amino end groups, and basic amino acids (i.e., lysine, histidine) that become part of the protein matrix of the cuticle [108] and are upregulated before ecdysis [108–110]. Chung et al. [111] recently found that, for initial cuticle formation, proPO may be participating actively in the initial cuticle formation in the blue crab, *C*. *sapidus* and granulocytes are responsible for proPO [83]. Not only does it appear that granular cells might be important in the initial stages of shell formation, but also during wound repair in the green crab, *Carcinus maenas* [112]. Furthermore, recent work by Heath-Heckman and McFall-Ngai [113] found that hemocytes synthesize chitin *de novo* in granular cells of *Euprymna scolopes*, the Hawaiian bobtail squid. Microscopic observation and genomic analyses on the eastern oyster, *Crassostrea virginica*, suggest that cell-mediated biomineralization through granular hemocytes could be occurring [105,114–115]. The work presented here suggests that SGC+GC pH_i_ under current conditions is more alkaline than the pH_e_. Furthermore there appears to be a relationship between the Ca concentration in the adults and the alkaline pH_i_ of these hemocytes, resulting in more calcium in the adult shells. Thus, granulocytes could be important in the initial stages of biomineralization, which raises the questions: (1) Does the pH_*i*_ of SGC+GC need to be higher for these processes to occur? (2) Is the SGC+GC pH_*i*_ related in any way to crystallization of calcite within the chitin layers of crustacean shells? (3) Could there be a combination of matrix-mediated and cell-mediated calcification of the carapace? (4) Does an alkaline SGC+GC pH_i_ maintain the ion balance so that less internal shell dissolution occurs? At this time this study cannot address any of these questions directly, but possible future research would involve studying the pH_*i*_, along with the presence of proPO activity, during different life stages of a crabs to determine if there is a change in the pH_*i*_ of the hemocytes and presence of granulocyte markers in the carapace of crustaceans through biochemical or molecular analyses.

Despite maintaining intracellular pH independent of external acidification, HC did differ in phagocytosis in response to acidification. The mean percentage of highly-phagocytic hemocytes in crabs held at pH 7.50 was more than double that found in crabs held at pH 8.09 or 7.80 (Fig 4). This stimulation of phagocytosis is consistent with a response to clear and recycle the large numbers of dead hemocytes in the hemolymph. Although phagocytosis was upregulated in these crabs it was not able to maintain low numbers of dead SGC+GC in circulation. This suggests a rapid rate of apoptosis and turnover of SGC+GC hemocytes in Tanner crabs in response to an acidified environment. If this is true, then it also implies that hematopoiesis of new SGC+GC hemocytes must have been higher in the crabs held at pH 7.50 as the total number of live cells was the same in all the treatments. A method to quantify apoptotic hemocytes in mollusks is used routinely at the Northeast Fisheries Science Center Laboratory; we consider it a high priority to test this hypothesis in the future. In the meantime, we can consider possible consequences of “disturbed” SGC +GC hematopoiesis and turnover in crabs resulting from exposure to environmental acidification.

The importance of the prophenoloxidase cascade in both immune defense and cuticle hardening has been underscored by Terwilliger and Ryan [116]. SGC +GC hemocytes are known to participate in this highly-regulated process [17]; therefore, disruption of “normal” functions in these cells could influence both immune defense and shell processes. Tanner et al. [117] showed that blue crabs, *C*. *sapidus*, exposed to an environmental pH of 7.0 had 16% lower phenoloxidase activity than crabs held at pH 7.8. In the present study, results indicate accumulation of dead SGC+GC hemocytes, possibly by apoptotic processes, at an environmental pH of 7.50. Although phagocytosis of HC was up-regulated at this pH, dead hemocytes still accumulated. Despite this accumulation of dead SGC+GC hemocytes, counts of live SGC+GC hemocytes were not diminished in crabs exposed to pH 7.50; this implies accelerated hematopoiesis to maintain a “standing stock” of presumably functional SGC+GC hemocytes. One can hypothesize that maturation of SGC+GC hemocytes (perhaps these two categories represent a maturation sequence) to full functionality [4] would be unable to maintain full phenoloxidase regulating function at pH 7.50. Measures of phenoloxidase activity in Tanner crabs held under acidified conditions could resolve this question. In the meantime, we have no evidence that this change in hemocyte profile–more dead SGC+GC hemocytes and more highly-phagocytic HC–is harmful to the crabs or diminishes immune defense functions. If this is a mechanism whereby crabs maintain immunological homeostasis, then it may come with an energetic cost that would be revealed by growth or reproductive differences.

Reproduction is an energetic-intensive process, which could propagate effects in the cellular defense system (hemocytes). Accordingly, based upon the principle of energy allocation, a gametogenic individual that is highly stressed would not be able to divert as much energy to other energetic-demanding processes (i.e., both phagocytosis, reproduction) [118,119]. Even though this study did not directly examine the energetic costs or the effects of spawning upon hemocyte function, we did note some potential correlations that suggest there was an energetic cost to maintain high levels of HC (phagocytic cells) and pH_i_. As noted, the number of dead hemocytes cells did vary significantly between treatments and there appears to be a negative relationship with the calcium concentration in the adults. This suggests that, as a consequence of regulating pH_i_ and pH_e_ in less-optimal physiological conditions, there may have been an energetic cost that allowed for Ca concentration in the shell to decrease and for a reduction in production of viable eggs. The mechanisms and the energetic costs need to be further explored.

In conclusion, this study found that an increase in *p*CO_2_ affected the cytological immune response and acid/base regulation of hemocytes of the Tanner crab. Furthermore, we found that there may be a relationship between hemocyte pH_i_, total egg numbers, the percentage of viable/non-viable eggs and Ca concentration in the adults. Immunological testing should be done with crustaceans and other marine organisms to examine any long-term effects that ocean acidification could have upon immunological functions. Further research is needed to examine if these changes in immunological functions will have a consequence for resistance to pathogens, as we begin to better understand how ocean acidification influences basic cellular immunological functions.
